# Buccal mucosal graft urethroplasty for anterior urethral stricture, experience from a low-income country

**DOI:** 10.1186/s12894-021-00918-0

**Published:** 2021-12-07

**Authors:** Sami Mahjoub Taha Awad, Musab Abdalla M. Ahmed, Yassin Mohamed Osman Abdalla, Mohammed El Imam M. Ahmed, Mohamed Daffalla-Awadalla Gismalla

**Affiliations:** 1 Department of Surgery, Faculty of Medicine, University of Gezira, Wad Medani, Gezira State Sudan; 2 Department of Urology, Gezira Hospital for Kidney Disease and Surgery, Wad Medani, Gezira State Sudan; 3 Sudan Medical Specialization Board, Khartoum, Sudan

**Keywords:** Urethroplasty, Anterior urethral stricture, Buccal mucosal graft urethroplasty, Urine retention

## Abstract

**Background/purpose:**

This study was conducted to present our experience in urethral mucosal graft urethroplasty to repair urethral stricture, as the first experience in our context.

**Methods:**

This is a prospective hospital-based study that had been designed to review management outcomes of buccal mucosal graft urethroplasty for anterior urethral stricture from January 2017 to January 2019.

**Results:**

The total number of involved patients was 60. The success rate was found to be 90% (n = 54), while 6 (10%) had a recurrence of stricture. Pain and pain combined bleeding from internal suture lines were the only early complication encountered in 50 (83.3%) and 2 (3.3%) patients, respectively. late complications occurred as follows 14 (23.3%) patients had UTI, 12 (20%) had wound infections, 8 (13.3%) had changes in ejaculation, and decrease in intensity of orgasm, and 6 (10%) had erectile dysfunction. One of the long-term complications was graft diverticulum in one case and was treated conservatively (in ventral on lay BMG).

**Conclusion:**

Improvement of the service in limited resources countries like Sudan and was reflected in the excellent outcome of BMG urethroplasty as treatment of anterior urethral stricture (success rate 90%).

## Backgrounds

Urethral stricture disease is a condition that affects 300 per 100,000 males and its surgical treatment can represent a challenge [1]. Several approaches including direct visual internal urethrotomy (DVIU) and anastomotic or augmentation urethroplasty based on the use of flaps and graft have been reported [2]. Barbagli described the dorsal onlay free graft urethroplasty in 1996 and since then graft urethroplasty has gained worldwide application for the treatment of penile and bulbar urethra strictures [3, 4].

Urethral reconstruction using a buccal mucosal graft (BMG) to substitute the urethral mucosa has become a well-established modality in the management of bulbar and penile urethral strictures, not amenable for excision and anastomosis. The various configurations of BMG urethroplasty have included ventral onlay, dorsal onlay, dorsal inlay via a ventral sagittal urethrotomy approach, dorsolateral onlay with one-sided mobilization of the urethra, combined dorsal plus ventral double mucosal grafts, two-stage repairs, and augmented anastomotic urethroplasty [5].

Although surgical short‐term visits tend to be the most predominant approach for overseas surgical assistance, workshops were a common method of overseas surgical support and if designed with a clear aim and locally relevant objectives, can be an excellent platform to augment surgical skills and meet specific learning needs [6].

Urethral stricture is one of the commonest obstructive uropthy which occur in urethra and presented to urologist in Sudan [7]. This is the first time to adopt BMG urethroplasty for management of anterior urethral stricture in our facility and this will affect the further outcome for patients and it will change the training and experience of the young surgeon and treatments provider. Further more, according to our data, this is the first written scientific work regarding BMG urethroplasty in Sudan. Previously it was managed with frequent DVIU and serial dilatation, whatever the stricture site and the length of the stricture, which was reflected in the poor outcomes [8].

In this study, we aimed to study the outcomes and complications for patients who underwent BMG urethroplasty at Gezira Hospital for Renal Disease and Surgery (GHRDS).

## Materials and methods

### Study design

This is a prospective hospital-based study designed to review the outcomes of buccal mucosal graft urethroplasty while treating anterior urethral stricture. The study was conducted from January 2017 to January 2019. The inclusion criteria in this study were: males patients above 15 years of age diagnosed with anterior urethral stricture (penile and bulbar), who underwent BMG with regular follow-up.

### Study area

Gezira Hospital for renal disease and Surgery is a governmental hospital located in Wad Medani city the Capital of Gezira state. It is the only referral hospital for the nearby state, Senar state, Blue Nile state, White Nile state, Gadarif state, and Kassala state. The total population cover is around 15 million. The hospital has a dialysis unit, nephrology department, surgical department, emergency department, operations room, radiological center, and laboratory center. The total number of beds is 100.

Sudan has been withered with the great devaluation of its currency and super inflation of its economy plus the four decades of civil wars. It was classify as low-income country.

The training of the health professionals was done by The SIU, IVUmed groups from India and Egypt conducted three training workshops in urethral strictures management including on hand surgery specially BMG in the hospital where four urologists are trained to shoulder these services in this hospital. They proved to have the sound capability and perfect skills.

### Perioperative care

Patients had been diagnosed with anterior urethral stricture after proper history, detailed examination, and urethrogram outpatient clinic visits. The operation was performed under spinal anesthesia until the time of graft harvesting then general anesthesia with nasal intubation for bilateral cheek graft or oral intubation for unilateral cheek graft, then the patient was put in a lithotomy position. Urethroscopy was done by semi-rigid ureteroscope (URS) size 6.5 FR tip to assess site, complete or partial obstruction, and the length of the stricture if only the guidewire was passed considered a complete obstruction, and if passable by URS considered as partial and then can be augmented. While the guidewire was placed in the urethra longitudinal incision was made along the sub-scrotal raphe and the bulbar urethral was explored, but the penile urethra usually invaginated below the scrotum in penile stricture.

The bulbospongiosum muscle was cut in the middle (nerve preservation), then the corpus spongiosum was mobilized and separated from the corpus cavernosum. The urethra was opened dorsally (the attached part of corpus cavernosum to corpus spongiosum) from a healthy-to-healthy urethral area under direct vision, then the BMG harvested by the following technique:

1. Using mouth retractor (Steinhauser retractor).
2. Identification of the parotid duct and marking the edges of the graft by povidone-iodine (marker pen is not available).
3. Hydro dissection by submucosal injection of saline mixed with adrenaline (every 2 ml diluted with 200 ml saline).
4. Then harvesting the BMG followed by de fating of the graft. The harvested graft was fixed dorsally to the corpus cavernosum by quilting stitch then the edges of the graft were sutured continuously to the urethra, corpus spongiosum, and corpus cavernosum bilaterally using 4/0 vicyl over silicon catheter of adequate size (14 or 16 FR). Then the harvested site most of the time is not sutured. Bleeding controlled only by external ice pack and coagulation. Lastly, the wound closed in layers. No drain was fixed in all operations.

### Post-operative follow-up

For perineal wound: removal of covering gauze on the second day and application of fusidic acid ointment for 1 week. For harvested BMG site: in the first day: internal packing and external ice packing, in the second day: non-spicy oral fluid, and the third day: soft diet.

Patient discharged from 4th to the 7th postoperative day on injectable third-generation cephalosporin and oral metronidazole for 7 days. The removal of the urethral catheter depends on the absence of extravasation on the per catheter urethrogram after 28 days.

Note: a suprapubic catheter is fixed as routine, then the urethral catheter was removed and the suprapubic catheter was tight for 3 days. After good micturition, for 3 days the suprapubic catheter was removed.

Long-term follow-up: after 3 months, the clinical assessment was conducted, and ascending urethrogram was performed. After 1 year, follow up also with clinical assessment and ascending urethrogram. Uroflowmetry was not functioning at the time of the study.

Ethical approval was obtained from approval acceptance to the hospital authority. Written and verbal consent were obtained from patients. Data used anonymously by using identity numbers instead of names is ordered to protect the patient’s identity and kept securely and in a separate file.

### Data collection

Data were collected from hospital records and patient’s file through structured questionnaires was used to collect data. Success and failure in terms of stricture recurrence, patient demographics, stricture etiology and anatomy, and the adverse outcomes and complications were recorded to determine risk factors for recurrent stricture and complications.

Data were analyzed by using a computer program Statistical Package for Social Sciences (SPSS V. 21.0). The analyzed data presented in tables and figures designed by Microsoft Excel 2007. Chi-square used as a significance test for categorical data respectively. The *P* value considered significant if < 0.05.

Ethical approval to conduct the study was obtained from the ethical committee of Faculty of Medicine, University of Gezira, all methods were carried out in accordance with relevant guidelines and regulations" in the methods section. A written consent was obtained from all subjects (under 18 years the concent obtained from a parent) to participate in the study and publish the data.

## Results

A total number of 60 patients was involved in the study. All of them underwent buccal mucosal graft (BMG) urethroplasty for anterior urethral stricture. Their mean age was 47 ± 15.3 years. Regarding the presenting complains, all the patients (n = 60; 100%) has weak stream, 40 (66.7%) patients had straining, 36 (60%) had burning micturition, 26 (43.3%) had acute urine retention and 16 (26.7%) patients had dribbling. Hypertension was the most common comorbidity presented in 26 (43.3%) of the patients and Diabetes Miletus in 16 (26.7%) patients. Mainly previous procedures were the main etiology of strictures as catheterization (n = 46; 76.7%). On the other hand, perineal pelvic injury was encountered in 14 (23.3%) patients and sexual transmitted disease (STDs) or non-gonococcal urethritis in 8 (13.3%) patients (Table 1). In the history of pervious surgery management, 22 (36.7%) patients underwent visual internal urethrotomy (DVIU) and 6 (10%) patients underwent urethral dilatation. Fifty-four (90%) of the patients underwent BMG urethroplasty for first time and 6 (10%) redo the operation (previous surgery was anastomotic or BMG).

**Table 1:** The stricture etiologies among patient's anterior urethral stricture who underwent buccal mucosal graft (BMG) urethroplasty (N = 60)

Catheterization	46	76.7
Cystoscopy	16	26.7
Pelvic surgery	8	13.3
Transurethral resection of the prostate (TURP)	6	10
Perineal pelvic injury	14	23.3
STDs or non-gonococcal urethritis	8	13.3

Regarding the stricture features, in majority of the patients 48 (80%) had stricture in bulbar site and 46 (76.7%) had stricture with more than 5 cm in length. Concerning to the operation characteristics, most of the cases underwent dorsal onlay operation (n = 54; 90%), had blood loss volume less than 500 ml (n = 58; 96.7%), had operation time from 1 to 2 h (n = 52; 86.7%) and used 16 FR catheter size (n = 44; 73.3%) (Table 2). Buccal mucosa harvesting site was mainly unilateral in 50 (83.3%) patients and bilateral in 10 (16.7%) patients (Table 2).

**Table 2:** Predictors for the outcome among patients of anterior urethral stricture who underwent buccal mucosal graft (BMG) urethroplasty (N = 60)

Variables	Success [n = 54]	Stricture recurrence [n = 6]	*P* value
Age			
< 20	2 (3.7%)	0 (0%)	0.857*
20–40	22 (40.7%)	2 (33.3%)	
41–60	24 (44.4%)	4 (66.7%)	
> 60	6 (11.1%)	0 (0%)	
Operation frequency			
First	52 (96.3%)	2 (3.7%)	0.020*
Redo	2 (33.3%)	4 (66.7%)	
Site of stricture			
Bulbar	48	0	0.000*
Penile	5	5	
Pan stricture	1	1	
Types of BMG urethroplasty			
Dorsal onlay	51 (94.4%)	3 (5.6%)	0.001**
Ventral onlay	2 (50%)	2 (50%)	
Anastomotic augmented dorsal onlay	1 (50%)	1 (50%)	
Expected blood loss			
< 500 ml	54 (93.1%)	4 (6.9%)	0.000**
> 500 ml	0 (0%)	2 (100%)	
Time of operation			
1–2 h	50 (96.2%)	2 (3.8%)	0.039**
> 2 h	4 (50%)	4 (50%)	
Size of catheter used			
14 FR	14 (85.7%)	2 (12.5%)	0.679*
16 FR	40 (90.9%)	4 (9.1%)	

*P value is not statistically significant

**P value is statistically significant

**Fig. 1:**
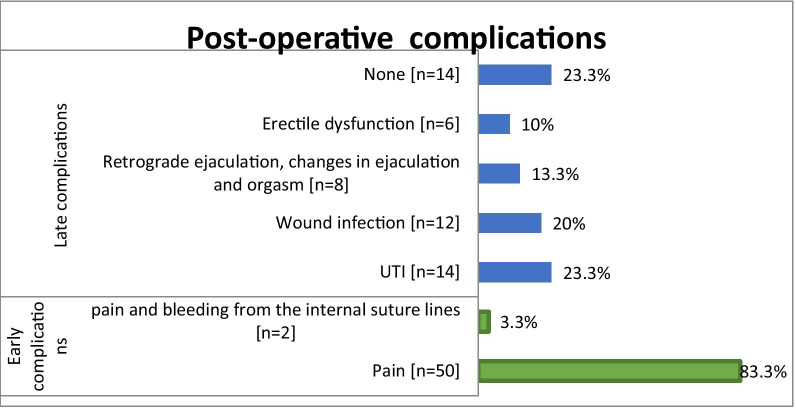
The postoperative complications among patients of anterior urethral stricture who underwent buccal mucosal graft (BMG) urethroplasty (N = 60)

The success rate was found to be 90% (n = 54), while 6 (10%) had recurrence of stricture (Fig. 1). Pain and pain combined bleeding from internal suture lines were the only early complication encountered in 50 (83.3%) and 2 (3.3%) patients, respectively. Late complications occurrence was found as follow, 14 (23.3%) patients had UTI, 12 (20%) had wound infections, 8 (13.3%) had changes in ejaculation, and decrease in intensity of orgasm, and 6 (10%) had erectile dysfunction (Fig. 1). One of the long-term complication was graft diverticulum in one case and was treated conservatively (in ventral onlay BMG).

According to the perioperative complications in harvesting site, pain presented in all patients (n = 60; 100%), 32 (53.3%) as moderate and 28 (46.7%) as minimal. Also, all the patients (n = 60; 100%) were normally able to drink, 40 (66.7%) patients were not able to eat initially, 6 (10%) had tightness and 4 (6.7%) patients had numbness. The median duration of pain relieving was 5 days, ranged from 3 to 14 days. Tightness was relieved after 2 days in 2 (33.3%) patients and after 3 days in 4 (66.7%) patients. numbness was relieved after one month in the 4 patients.

Peri-catheter urethrogram showed no extravasations in 56 (93.3%) patients and extravasations in 4 (6.7%) patients, so the catheter was kept for another 2 weeks. also, post-operative ascending urethrogram (3rd and 12th month) was normal 53 (88.3%), abnormal (filing defect) in 6 (10%) patients and one patient (1.7%) had diverticulum.

The volume of blood loss was significantly affected the outcomes (*P* value = 0.000) as the success rate was 93.1% in those lost less than 500 ml of blood compared to no patients of those lost more than 500 ml of blood.The time of operation was significantly affected the outcomes (*P* value = 0.039) as the success rate was high in patients with operation time from 1 to 2 h (96.2%) more than those with operation time more than 2 h (50%). The success or outcomes of BMG urethroplasty was not significantly affected by the sizes of catheter used (*P* value = 0.679).

## Discussion

Humby was the first to use buccal mucosa for urethral reconstruction in 1941 for hypospadias repair [9]. Because buccal mucosal grafts have advantages over other grafts, they have been popular since the 1990s. Open urethroplasty is the gold standard treatment for urethral strictures, but it is not a routine operation for a general urologist. Since 1993 El-Kasaby et al. reported the first experience with buccal mucosa urethroplasty for treatment of penile and bulbar urethral strictures [10].

In the present study, most of the patients 48 (80%) had a stricture in the bulbar site. This is in concordance with literature as up to 90% of urethral strictures were in the anterior urethra, and approximately two-thirds (75%) of them occur in the bulbar urethra [1, 11, 12]. The common etiologies of urethral strictures in this study was catheterization (76.7%), while, perineal pelvic injury found in 14 (23.3%) patients and STDs or non-gonococcal urethritis in 8 (13.3%) patients. However, in the study of Mohamed et al.; the cause of stricture was idiopathic in 47, inflammatory in 15, lichen-sclerosis in 26, and post failed hypospadias repair in 35 patients [13]. In the study of Kamyar et al., the etiology of the urethral stricture was sexually transmitted disease (STD) in (14.53%), lichen sclerosis in (12.82%), a trauma in (12.82%), catheterization in (11.11%), transurethral resection (TUR) in (5.13%), and unknown in (43.59%) [14].

In this study, the vast majority of the patients underwent dorsal onlay operation (n = 54; 90%). And this can be explained by the dorsal onlay procedure are a more stable and reliably well‐vascularized graft bed, and less possibility of sacculation our results were similar to Figler et al. [15]. The time of catheter removal was fewer than 4 weeks (22–26 days) in 12 patients (20%) due to uncontrolled infection, fortunately, post operatively was good. The current study reported, the overall success rate was 90% (n = 54 patients). This rate was similar to the studies of Tarun et al. who reported the rate of 91% [6], Mohamed et al. who reported a success rate of 91.1% [11], Kamyar et al. who reported the tare of 93.3% [14]. And the lowest success rates reported by Marco et al. (81%) [16] and Yalcinkaya et al. (70%) [17] and Guido et al. (80%) [18]. Barbagli described the dorsal onlay graft urethroplasty in 1996 in 12 patients, reporting a successful outcome in all patients [19]. Morey and McAninch reported similar results in 13 patients who underwent ventral onlay BMG urethroplasty for anterior urethral stricture [20]. Barbagli more recently reported the 80.2% long-term success rate for dorsal onlay bulbar urethroplasty using BMG [19]. Variations to this approach have been reported by Kulkarni, with success rates up to 92% [21]. Asopa described the dorsal inlay free graft urethroplasty and with this technique, excellent results up to 87% of success rate have been reported [22]. The variations in the success rates might be attributed to the difference in geographical areas, sampling as well as the types of BMG urethroplasty.

Not surprisingly, the pain was presented in all the patients' post-operation, in addition to urinary tract infection (23.3%) and superficial wound infection (20%) were the major late complications, the high incidence of infection (UTI and superficial wound infection) can be explained by the absence of urine culture and sensitivity test in this study as a prerequisite before surgery, which might reduce this rate especially most of the patients had an increase in post voiding residual urine volume, which predispose to UTI. In the study of Marco et al., the most frequent complications recorded were urinary fistula (3.1%), graft contracture (3.1%), and graft failure (3.1%) [16]. While Tarun et al. reported wound infection was the main postoperative complication [6]. The study of Mohamed et al. reported patients complicated by fistula (5.7%), wound infection (1.9%), and meatal stenosis (2.9%) [13].

Regarding the complication in the harvesting site, all the patients (n = 60) had pain, and two-thirds (66.7%) were unable to eat initially, 6 (10%) had tightness and 4 (6.7%) patients had numbness. This compatible with the findings of Wood et al. study [23]. The results showed the age of the patients was not significantly affected the outcomes of BMG urethroplasty. This finding was in agreement with the study of Yalcinkaya et al. [17] and Biswajit et al. [24]. Interestingly, the success rate of BMG urethroplasty was significantly high among the patients who underwent the procedure for the first time (93.3%) more than those with redo (33.3%) (*P* value = 0.020) because of less fibrous tissue in comparison with redo urethroplasty. This was in agreement with a review of Lindsay et al. who reported the failure rate of urethroplasty increases substantially with repeated procedures [25].

Also, showed the success rate was 100% among the patients with stricture in bulbar, 50% in a pan, and penile stricture (*P* value = 0.000). These findings were comparable to the study of Marco et al. [16] and Yalcinkaya [17]. The success rate was 100% among the patients with stricture length less than 5 cm whilst in those with stricture length more than 5 cm the success rate was 87% (*P* value = 0.043) the restructure was found in the proximal and distal end of the graft anastomosis. The current results were consistent with the studies of Marco et al. (14) [16] and Yalcinkaya et al. [17]. The type of operation has significantly affected the outcomes (*P* value = 0.001) as the success rate was high in dorsal onlay (92.9%) more than ventral onlay (50%). A recent review described the outcome of 35 studies of the success rate of the dorsal onlay urethroplasty in a total of 934 patients and reported that with an average follow-up of 42.2 months the average success rate was 88.4% [26].

The volume of blood loss has significantly affected the outcomes (*P* value = 0.000) as the success rate was 93.1% in those who lost less than 500 ml of blood compared to no patients of those who lost more than 500 ml of blood. The time of operation has significantly affected the outcomes (*P* value = 0.039) as the success rate was high in patients with operation time from 1 to 2 h (96.2%) more than those with operation time of more than 2 h (50%). And remarkably, this study showed the success rate was 100% among the patients with unilateral buccal mucosal sites whilst in those with bilateral buccal mucosal sites the success rate was 40% (*P* value = 0.002). This observation was comparable to the study of Irekpita et al. [27].

## Conclusion

Inside training by expert international trainers is an excellent method of improvement of the service in limited resources countries like Sudan and that was reflected on the excellent outcome of BMG urethroplasty as treatment of anterior urethral stricture (success rate 90%).

## Data Availability

The data and information are available with the correspondence author if requested.

## Abbreviations

BMG: Buccal mucosal graft
STDs: Sexual transmitted diseases
GHKDS: Gezira Hospital for Kidney Disease and Surgery

## References

[1] Stein DM, Thum DJ, Barbagli G (2013). A geographic analysis of male urethral stricture aetiology and location. BJU Int.

[2] Mangera A, Osman N, Chapple C (2016). Evaluation and management of anterior urethral strictre disease. F1000Res.

[3] Barbagli G, Selli C, Tosto A (1996). Dorsal free graft urethroplasty. J Urol.

[4] Chapple C, Andrich D, Atala A (2014). SIU/ICUD consultation on urethral strictures: the management of anterior urethral stricture disease using substitution urethroplasty. Urology.

[5] Tarun D, Amit K, Harohalli K (2016). Management of recurrent anterior urethral strictures following buccal mucosal graft-urethroplasty: a single center experience. Urol Ann..

[6] Shrime MG, Sleemi A, Ravilla TD (2015). Charitable platforms in global surgery: a systematic review of their effectiveness, cost-effectiveness, sustainability, and role training. World J Surg.

[7] El Imam M, Omran M, Nugud F, Elsabiq M, Saad K, Taha O (2006). Obstructive uropathy in Sudanese patients. Saudi J Kidney Dis Transplant.

[8] Mansour MO, Aboagla YA, Taha SM, Khalid KE (2013). Urethral stricture; etiology, presentation, complications, and outcome of management at Gezira State, Central Sudan. Gezira J Health Sci.

[9] Humby G (1941). A one-stage operation for hypospadias. Br J Surg.

[10] El-Kasaby AW, Fath-Alla M, Noweir AM, El-Halaby MR, Zakaria W, El-Beialy MH (1993). The use of buccal mucosa patch graft in the management of anterior urethral strictures. J Urol.

[11] Fenton AS, Morey AF, Aviles R, Garcia CR (2005). Anterior urethral strictures: etiology and characteristics. Urology.

[12] Palminteri E, Berdondini E, Verze P, De Nunzio C, Vitarelli A, Carmignani L (2013). Contemporary urethral stricture characteristics in the developed world. Urology.

[13] Mohamed S, Shady S, Eid E, Atef B (2019). Outcome of staged buccal mucosal graft for repair of long segment anterior urethral stricture. BMC Urol.

[14] Kamyar T, Alireza G (2014). Dorsally placed buccal mucosal graft urethroplasty in treatment of long urethral strictures using one-stage transperineal approach. Int Sch Res Not.

[15] Figler BD, Malaeb BS, Dy GW, Voelzke BB, Wessells H (2013). Impact of graft position on failure of single-stage bulbar urethroplasties with buccal mucosa graft. Urology.

[16] Marco S, Neha S, Sachin M, Mahreen H (2017). Buccal mucosal graft urethroplasty in men—risk factors for recurrence and complications: a third referral centre experience in anterior urethroplasty using buccal mucosal graft. Transl Androl Urol.

[17] Yalcinkaya F, Zengin K, Sertcelik N, Yigitbasi O (2015). Dorsal onlay buccal mucosal graft urethroplasty in the treatment of urethral strictures—does the stricture length affect success?. Adv Clin Exp Med..

[18] Guido B, Giorgio G, Enzo P (2006). Anastomotic fibrous ring as cause of stricture recurrence after bulbar onlay graft urethroplasty. J Urol.

[19] Barbagli G, Kulkarni SB, Fossati N (2014). Long-term followup and deterioration rate of anterior substitution urethroplasty. J Urol.

[20] Morey AF, McAninch JW (1996). Technique of harvesting buccal mucosa for urethral reconstruction. J Urol.

[21] Kulkarni S, Barbagli G, Sansalone S (2009). One-sided anterior urethroplasty: a new dorsal onlay graft technique. BJU Int.

[22] Asopa HS, Garg M, Singhal GG (2001). Dorsal free graft urethroplasty for urethral stricture by ventral sagittal urethrotomy approach. Urology.

[23] Wood D, Allen S, Andrich D (2004). The morbidity of buccal mucosal graft harvest for urethroplasty and the effect of nonclosure of the graft harvest site on postoperative pain. J Urol.

[24] Biswajit DM, Rao RP, Acharya L (2007). Dorsal onlay buccal mucosal graft urethroplasty in long anterior urethral stricture. Int Braz J Urol.

[25] Lindsay A, Jack W, Benjamin N (2014). Male urethral strictures and their management. Nat Rev Urol.

[26] Mangera A, Patterson JM, Chapple CR (2011). A systematic review of graft augmentation urethroplasty techniques for the treatment of anterior urethral strictures. Eur Urol.

[27] Irekpita E, Esezobor E (2016). Buccal mucosal graft urethroplasty for proximal bulbar urethral stricture: a revisit of the surgical technique and analysis of eleven consecutive cases. Niger Med J.

